# Whole exome sequencing of a single osteosarcoma case—integrative analysis with whole transcriptome RNA-seq data

**DOI:** 10.1186/s40246-014-0020-0

**Published:** 2014-12-11

**Authors:** Ene Reimann, Sulev Kõks, Xuan Dung Ho, Katre Maasalu, Aare Märtson

**Affiliations:** Department of Pathophysiology, University of Tartu, 19 Ravila Street, Tartu, 50411 Estonia; Department of Reproductive Biology, Estonian University of Life Sciences, 64 Kreutzwaldi Street, Tartu, Estonia; Department of Traumatology and Orthopaedics, University of Tartu, 8 Puusepa Street, Tartu, Estonia; Department of Oncology, Hue University of Medicine and Pharmacy, 6 Ngo Quyen Street, Hue, Vietnam; Traumatology and Orthopaedics Clinic, Tartu University Hospital, 8 Puusepa Street, Tartu, Estonia

**Keywords:** Osteosarcoma, Whole exome sequencing, Integrative analysis

## Abstract

**Background:**

Osteosarcoma (OS) is a prevalent primary malignant bone tumour with unknown etiology. These highly metastasizing tumours are among the most frequent causes of cancer-related deaths. Thus, there is an urgent need for different markers, and with our study, we were aiming towards finding novel biomarkers for OS.

**Methods:**

For that, we analysed the whole exome of the tumorous and non-tumour bone tissue from the same patient with OS applying next-generation sequencing. For data analysis, we used several softwares and combined the exome data with RNA-seq data from our previous study.

**Results:**

In the tumour exome, we found wide genomic rearrangements, which should qualify as chromotripsis—we detected almost 3,000 somatic single nucleotide variants (SNVs) and small indels and more than 2,000 copy number variants (CNVs) in different chromosomes. Furthermore, the somatic changes seem to be associated to bone tumours, whereas germline mutations to cancer in general. We confirmed the previous findings that the most significant pathway involved in OS pathogenesis is probably the WNT/β-catenin signalling pathway. Also, the IGF1/IGF2 and IGF1R homodimer signalling and TP53 (including downstream tumour suppressor gene *EI24*) pathways may have a role. Additionally, the mucin family genes, especially *MUC4* and cell cycle controlling gene *CDC27* may be considered as potential biomarkers for OS.

**Conclusions:**

The genes, in which the mutations were detected, may be considered as targets for finding biomarkers for OS. As the study is based on a single case and only DNA and RNA analysis, further confirmative studies are required.

**Electronic supplementary material:**

The online version of this article (doi:10.1186/s40246-014-0020-0) contains supplementary material, which is available to authorized users.

## Introduction

Osteosarcoma (OS) is a most prevalent primary malignant bone tumour and mostly occurs in children and adolescents—75% of patients with OS are 15 to 25 years old. The etiology is unknown; however, a genetic predisposition has been suggested [1,2]. Reviewed in [3], these tumours have high potential to metastasize and are one of the most frequent causes of cancer-related deaths. The survival rate increased up to 70% after chemotherapy became available [4]. However, no further improvements have been made in the last decades in terms of survival. Thus, the survival plateau forces scientists to look for new biomarkers (diagnostic, disease monitoring, response, resistance markers, drug targets), which could lead to, i.e. applying new therapeutic agents. While OS is rare and very heterogeneous (inter-patient, inter-tumour and intra-tumour heterogeneity), the clinical study progress is slow; thus, the preclinical studies are vital. Furthermore, finding the biomarkers and detecting the potential targets for new drugs are essential to improve the present situation.

There are several next-generation sequencing (NGS) and genome-wide association studies (GWAS) about OS, which associate different genes and pathways with pathogenesis of OS [5-7]. With whole exome sequencing (WES) and whole genome sequencing (WGS) studies, *TP53*, *PTEN* and *PRB2* are found to be mutated in significant frequency [5]. High mutation rate in *TP53* has also demonstrated in OS cell lines. Additionally, deletion of *CDKN2A*/*B* locus and amplification of *MDM2* were detected [8]. With GWAS studies, a single nucleotide variant (SNV) in *GRM4* was detected as potential biomarker for OS [7]. Gene expression studies reveal that, i.e. WNT inhibitory factor (*WIF1*) has a loss of expression in OS cell lines [9]; however, we found in our previous work that the expression has increased significantly [10]. Thus, as demonstrated, the expression pattern of WNT pathway genes in different OS cases may not be similar. When correlating the expression patterns of miRNA/mRNA pairs, miRNAs regulating *TGFBR2*, *IRS1*, *PTEN* and *PI3K* have been detected [11]. In addition, several serine/threonine kinases (mechanistic target of rapamycin (mTOR)) or tyrosine kinases (SRC, IGF1R, PDGFR, KIT) are considered as targets in OS treatment [3,12,13].

When observing the related pathways, the WNT/β-catenin pathway is one of the most thoroughly studied among bone malignancies. For example, the tumour growth is regulated through this pathway and the overexpression of *BMP9* suppresses its activity [14,15]. Furthermore, PI3K/AKT/mTOR signalling pathway was brought forward as a potential target for therapy, and also, pathways associated to TP53 may be altered [5]. Hypoxia-HIF-1α-CXCR4 pathway plays a crucial role during the migration of human osteosarcoma cells [16]. These are just a few examples—the network of associated genes and pathways is complex.

OS has a very unstable genome—it may contain aberrant number of chromosomes, and in most cases, these chromosomes display major structural abnormalities including amplification, deletions and translocations. For example, several studies have demonstrated the gain of chromosomal arms 6p, 8q and 17p in the case of OS [17,18]. To be more precise, i.e. *VEGFA* amplification and *LSAMP* deletion have been detected in OS [19,20]. Thus, it is suggested that genomic instability is linked to the development of this tumour [18,21-23]. Furthermore, the genomic aberrations are more frequent in metastases than in primary tumours [24]. The genes responsible for cell cycle regulation are suggested to be associated to DNA breakage and genomic instability, i.e. *CDC5L* overexpression and mutations in *TP53* gene are correlated to the high genomic instability in OS [23,25].Moreover, the chromothripsis event is characteristic to OS—it generates new fusion products. This may explain the sudden onset of OS and the complexity and heterogeneity of OS genome [26]. All these changes make it difficult to find biomarkers suitable for targeting OS, as there are so many different subtypes.

In the present work, we analysed the whole exome of the tumorous and non-tumour bone tissue from the same patient with osteosarcoma. We used next-generation sequencing to study how the coding region of the tumour genome has altered. Additionally, we analysed together the WES genotyping and RNA expression data (from our previous RNA-seq analysis).

## Materials and methods

### Subject

The protocols and informed consent form used in this study were approved by the Ethical Review Committee on Human Research of the University of Tartu. The patient signed a written informed consent, which also includes the acceptance of the report to be published. A 16-year-old Caucasian male patient with an OS diagnosis was studied. In more detail, the patient became ill with complaints of pain in the left knee area. History of trauma was missing, and GP administered painkillers and vitamins. After 6 months, the patient returned to GP with complaints of pain, swelling and dysfunction in the left distal femur and knee area. The swelling line was observed in the left femoral distal region, and the area was thicker and painful to touch. No changes in skin colour were detected. The X-ray investigation showed additional shading and structural change in the distal part of the left femur. For detailed investigation, the MRI was performed and as a result, malignant process was suspected. Patient was hospitalized, and bone biopsy was taken for histological investigation. The diagnosis of osteosarcoma was confirmed. Chemotherapy for osteosarcoma started by Scandinavian Sarcoma Group (SSG) XIV treatment protocol. The patient responded well to the therapy—the histological analysis confirmed the necrotic tissue in tumour. After 3 month of chemotherapy, surgical removal of tumour (distal part of femoral bone with knee joint) and replacement of the knee and the lower part of the femur with megaprosthesis was performed. Pathologist confirmed that resection line was without tumour cells and OS was referred as NAS (Not Further Specified). After the patient had recovered from surgery, the SSG XIV chemotherapy treatment protocol was followed. Materials for this study were collected from the surgically removed tissue.

### Exome sequencing

The genomic DNA (gDNA) was extracted from two bone samples from different locations—one sample from tumour area and another sample from the uninvolved normal bone tissue as a control. For gDNA extraction, the tissue was homogenized applying liquid nitrogen and a mortar, and after that, the PureLink Genomic DNA kit (Life Technologies Corp., Carlsbad, CA, USA) was used according to manufacturer’s protocol. The Target Seq Exome Enrichment System and SOLiD 5500 barcoded adaptors (Life Technologies Corp., Carlsbad, CA, USA) were used to prepare the libraries. The SOLiD 5500xl platform and paired-end DNA sequencing chemistry (75 bp forward and 35 bp reverse direction) were applied to sequence the samples.

### The data analysis

Offline cluster was used for data processing and analysis. For bioinformatic analysis, LifeScope version 2.5 was applied. LifeScope performed colour space mapping and pairing. Tertiary analysis consisted of SNV discovery (diBayes algorithm) and detection of small indels. Hg19 (GRCh37.p13) was used as a reference, and before mapping, the multifasta file was verified in order to increase the mapping quality.

The SNVs and small indel .gff3 files were used as input in ANNOVAR software (AS; www.openbioinformatics.org/annovar/) [27] and Ingenuity Variant Analysis (IVA; http://www.ingenuity.com) QIAGEN, Redwood City, MD, USA) software. Applying refGene hg19, dbSNP135 and dbCOSMIC67 databases, AS annotated and predicted the effects of SNVs and small indels we detected in our study samples. AS also provides other prediction tools in order to get prediction scores (PolyPhen-2, SIFT, ljb2 etc.) [28-30]. Comparative distribution of SNVs and small indels between different samples was performed with Galaxy software bundle [31,32]. IVA provided tools to annotate SNVs and small indels, which may be associated to cancer. The tumour and control samples were compared, and the lists for diseases, processes and pathways related to cancer were received as output.

The .bam and .bai files were used as input in CEQer software (CS) (www.ngsbicocca.org/html/ceqer.html), which is a tool for analysing copy number variants (CNVs) and loss of heterozygosity (LOH).

About the RNA-seq data analysis, please see our previous article, where we used the bone samples from the same patient [10].

## Results

For comparing the tumour tissue and non-tumour tissue (control tissue) from the same individual, different approaches were applied. After mapping the data to a reference genome, we used several tools to perform the tertiary analysis.

### Sequencing statistics from LifeScope software

In the case of the tumour tissue, over 130 million (58%) mappable reads were in target and the enrichment fold was 48%. Eighty-five percent of the detected targets were covered over 20 times, and the average coverage was 185.5. In the case of the control tissue, over 154 million (61%) mappable reads were in target and the enrichment fold was 51%. Eighty-three percent of the detected targets were covered over 20 times, and the average coverage was 157.

### SNVs, small indels and CNVs

1. **Results from ANNOVAR software**

Using refGene hg19 database, AS was able to annotate 37,990 SNVs and 1,484 small indels. In the case of SNVs, we considered the data reliable, if the coverage was over 20; thus, 25,914 SNVs remained. In the case of SNVs, there were 23,767 germline mutations (9,067 in homozygous form and 14,700 in heterozygous form) and 2,147 somatic mutations (in the tumour tissue—116 in homozygous form and 2,031 in heterozygous form) (Table 1, Additional file 1). Furthermore, there were 896 germline small indels (278 in homozygous form and 618 in heterozygous form) and 588 somatic indels (in the tumour tissue—177 in homozygous form and 411 in heterozygous form).

**Table 1:** **The numbers of SNV and small indel findings received from data analysis with ANNOVAR software**

	**Germline mutations**		**Somatic mutations**			
	**Homozygous: non-reference**	**Heterozygous**	**Homozygous in tumour**	**Heterozygous in tumour**	**Homozygous in tumour**	**Heterozygous in tumour**
			**Heterozygous in control**	**Homozygous in control**	**Homozygous (reference) in control**	**Homozygous (reference) in control**
SNVs						
Altogether	9,067	14,700	48	237	68	1,794
Exonic (includes ncRNA)	5,244	8,702	21	103	29	967
Nonsynounymous	2,435	4,035	15	52	18	500
Stopgain	6	50	0	2	0	7
Stoploss	2	5	0	0	0	1
Splicing (includes exonic)	11	20	0	0	0	2
Intronic (includes ncRNA)	3,091	4,846	22	111	35	681
5′ UTR and 3′ UTR	515	797	3	19	1	91
Downstream and upstream	51	76	1	2	1	13
Intergenic	155	259	1	2	2	40
Small indels						
Altogether	278	618	89	75	88	336
Exonic (includes ncRNA)	33	99	14	11	2	29
Frameshift	9	30	4	4	1	12
Stopgain	0	1	0	0	0	0
Splicing (includes exonic)	4	16	0	4	1	3
Intronic (includes ncRNA)	212	419	64	51	75	270
5′ UTR and 3′ UTR	25	73	10	7	8	24
Downstream and upstream	1	2	0	1	1	6
Intergenic	3	9	1	1	1	4

Applying dbSNP135, we were able to annotate 5,281 SNVs and 239 small indels. With dbCOSMIC67, we annotated 2,569 SNVs and 59 small indels—none of these were noted to be associated to bone cancer. Applying ljb2 database, we found 469 SNVs to potentially cause a disease (average ljb2 score over 0.918), including 31 germline mutations and 4 somatic mutations (*ESX1*: c.A578G/p.K193R; *CDC27*: c.A17G/p.E6G; *TMEM120B*: c.G274A/p.D92N; *TMEM131*: c.C3947T/p.P1316L) in homozygous form in the tumour tissue.

2) **Results from Ingenuity Variant Analysis software**

Altogether, 207 cancer driver variants (CD-SNVs) were found in 123 genes according to IVA (Additional file 2). Fourteen CD-SNVs potentially gain and 186 lose the gene function. Only seven SNVs may have no drastic effect on gene function in the tumour tissue. Furthermore, according to IVA, none of these 207 SNVs affect the gene functionality in the control tissue. Thirteen of the CD-SNVs were homozygous in the tumour tissue (Table 2). There were no cancer-associated homozygous mutations present in the control tissue; thus, the homozygous CD-SNVs in the tumour tissue are all somatic.

**Table 2:** **The somatic cancer driver SNVs and small indels found in data analysis with Ingenuity Variant Analysis software**

**Gene symbol**	**Chr number**	**Position**	**REF/ALT**	**Tumour zygosity**	**Effect on function**	**Control zygosity**	**Effect on function**	**dbSNP**	**SIFT function**	**Polyphen function**	**Transcript ID**	**Nucleotide change**	**Amino acid change**	**Gene region**	**Translation impact**
SNVs															
RGPD3 (includes others)	2	110585652	A/G	1/1	Loss	0/0	Normal		Damaging	Benign	NM_001037866.1,	c.2393A > G	p.E798G	Exonic	Missense
											NM_001123363.3,				
											NM_005054.2,				
											NM_032260.2				
PRDM9	5	23527251	C/T	1/1	Loss	0/0	Normal		Tolerated	Probably damaging	NM_020227.2	c.2054C > T	p.T685I	Exonic	Missense
FOXK1	7	4722436	A/G	1/1	Loss	0/0	Normal		Damaging	Benign	NM_001037165.1	c.497A > G	p.N166S	Exonic	Missense
CCZ1/CCZ1B	7	6841033	T/A	1/1	Loss	0/1	Normal		Tolerated		NM_198097.3	c.1228A > T	p.M410L	Exonic	Missense
PLAT^a^	8	42044965	G/A	1/1	Normal	0/0	Normal	2020921	Tolerated	Benign	NM_033011.2/	c.352C > T/	p.R118W/	Exonic	Missense
											NM_000930.3	c.490C > T	p.R164W		
AGTPBP1^a^	9	88292495	C/T	1/1	Loss	0/1	Normal		Tolerated	Benign	NM_015239.2	c.292G > A	p.G98R	Exonic	Missense
SARDH	9	136597592	T/C	1/1	Loss	0/0	Normal	149002589	Tolerated	Benign	NM_001134707.1,	c.463A > G	p.I155V	Exonic	Missense
											NM_007101.3				
FAH	15	80472526	C/T	1/1	Normal	0/1	Normal	11555096	Damaging	Probably damaging	NM_000137.2	c.1021C > T	p.R341W	Exonic	Missense
CDC27	17	45266522	T/C	1/1	Loss	0/0	Normal	62077279	Damaging	Probably damaging	NM_001114091.1,	c.17A > G	p.E6G	Exonic	Missense
											NM_001256.3				
SBF1^a^	22	50893287	T/C	1/1	Loss	0/1	Normal	200488568	Tolerated	Benign	NM_002972.2	c.4768A > G	p.T1590A	Exonic	Missense
LRRC37A3^a^ (includes others)	17	44632540	T/C	1/1	Gain	0/0	Normal	144051917	Activating	Benign	NM_001006607.2	c.4882 T > C	p.W1628R	Exonic	Missense
ARL17A	17	44632540	T/C	1/1	Gain	0/0	Normal	144051917	Activating	Benign	NM_001113738.1/	c.*2182A > G/	-/	3'UTR/	
											NM_016632.2	c.259 + 15585A > G	-	Intronic	
LILRB3	19	54725835	G/C	1/1	Gain	0/0	Normal	201948566	Activating	Benign	NM_001081450.1,	c.523C > G	p.R175G	Exonic	Missense
											NM_006864.2				
Small indels															
CTCFL	20	56073500	(N)103/T	1/1	Loss	0/0	Normal				NM_001269041.1/	c.*4_*105del(N)103/		3′ UTR/	
											NM_001269043.1/	c.1988 + 8_1988 + 109del(N)103/		Intronic/	
											NM_001269040.1/	c.*4_*105del(N)103/		3′ UTR/	
											NM_001269042.1/	c.*4_*105del(N)103/		3′ UTR/	
											NM_080618.3/	c.*4_*105del(N)103/		3′ UTR/	
											NM_001269046.1	c.*4_*105del(N)103		3′ UTR	
PRR23C	3	138763627	GTGC/G	1/1	Loss	0/1	Normal	63140560			NM_001134657.1	c.-168_-166delGCA		5′ UTR	
CDCA7L	7	21941867	CTTAG/C	1/1	Loss	0/0	Normal				NM_001127371.2/	c.*69_*72delCTAA/		3′ UTR/	
											NM_001127370.2/	c.*69_*72delCTAA/		3′ UTR/	
														3′ UTR	
											NM_018719.4	c.*69_*72delCTAA			
ALK	2	29416029	G/GATTG	1/1	Loss	0/0	Normal				NM_004304.4	c.*60_*61insCAAT		3′ UTR	
DSPP	4	88537081	CAGCAGCAAT/C	0/1	Loss	0/0	Normal				NM_014208.3	c.3268_3276delAGCAGCAAT	p.S1090_N1092del	Exonic	In-frame
RELA	11	65422086	CTC/CTGTAGT	0/1	Loss	0/0	Normal				NM_001145138.1/	c.1408delGinsACTAC/	p.E470fs*19	Exonic/	Frameshift/
											NM_021975.3/	c.1417delGinsACTAC/		Exonic/	Frameshift/
											NM_001243984.1/	c.1210delGinsACTAC/		Exonic/	Frameshift/
											NM_001243985.1	c.1216-108delGinsACTAC		Intronic	-

^a^The expression pattern of these genes has changed in the tumour tissue compared to that in the control tissue.

According to IVA, six cancer-associated small indels were found (Table 2). Four of them are homozygous and two are heterozygous in the tumour tissue—the effect is most probably the loss of gene function. These indels are predicted to have no effect in the control tissue.

In most of the genes brought front by IVA, one CD-SNV was found in coding region in heterozygous form. However, some of the genes have more CD-SNVs in coding regions: *MUC4* had even 22, *ZNF717* had 8, *CTBP2* had 7 and *OR4C3* had 5 CD-SNVs, whereas these were not present in the control tissue (data not shown). When observing from a slightly different angle—the gene complexes, we can see that the mucin complex has the highest significance—three genes and 27 CD-SNVs are considered (Table 3). There are also other gene complexes, which are potentially associated to cancer processes, and in different complexes, the CD-SNVs are either somatic or germline (Table 3).

**Table 3:** **The gene complexes which are potentially associated to cancer processes**

**Complex name**	***p*** **value**	**Number of genes associated**	**Number of variances found**	**Tumour tissue**	**Control tissue**
Mucin	9.54E-05	3: *MUC2*, *MUC4*, *MUC6*	27	1	0
Bcl9-Cbp/p300-Ctnnb1-Lef/Tcf	2.46E-03	2: *CREBBP*, *TCF3*	2	1	0
Sox	4.55E-03	2: *SOX7*, *SOX10*	2	1	0
Cholesterol monooxygenase (side-chain-cleaving)	1.06E-02	1	1	1	0
CYP11A	1.06E-02	1	1	1	0
Sarcosine dehydrogenase	1.06E-02	1	1	1	0
Ctbp	1.59E-02	1	7	1	0
Cbp/p300	1.59E-02	1	1	1	0
Dimethylglycine dehydrogenase	1.59E-02	1	1	1	0
DRD1/5	1.59E-02	1	1	1	0
MAGI	2.64E-02	1	2	1	1
Magi-Pten	3.68E-02	1	2	1	1
Fumarylacetoacetase	1.06E-02	1	1	1	1

There are both somatic and germline cancer driver SNVs found in the tumour and control tissues.

In the case of cancer-associated small indels, the statistically most significant results were with complexes related to *RELA* gene—NFKB1-RELA and RELA-REL complexes both had *p* value 7.56E-4.

IVA provided the first 100 cancer-associated processes and diseases related to CD-SNVs and small indels. Seventy-three genes and 135 CD-SNVs were found associated to process named as “disorder of genitourinary system” (Table 4). These findings were present in both the tumour and control tissues. There were also two processes associated to bone “myelopoiesis of bone marrow” (associated genes *NPM1*, *RARA*) and “quantity of trabecular bone” (associated genes *CREBBP*, *SMO*)—these findings were present only in the tumour tissue. In the case of small indels, all the findings were somatic and *ALK* and *RELA* genes were associated to “outgrowth of bone marrow cells” and “inflammatory response of bone marrow-derived macrophages”, respectively.

**Table 4:** **The cancer-associated processes detected by IVA**

**Process name**	***p*** **value**	**Number of genes associated**	**Number of variances found**	**Tumour tissue**	**Control tissue**
CD-SNVs					
Disorder of genitourinary system	9.05E-14	73	135	1	1
Cell biology	4.08E-04	69	132	1	1
Cell signalling	3.83E-03	25	31	1	1
Morphology of body region	2.55E-03	23	24	1	1
Abnormal morphology of cells	1.73E-03	18	19	1	1
Abnormal morphology of body cavity	6.17E-04	17	18	1	1
Morphology of body cavity	1.36E-03	17	18	1	1
Morphology of cardiovascular system	5.80E-04	13	14	1	1
Abnormal morphology of cardiovascular system	7.22E-04	12	13	1	1
Abnormal morphology of thoracic cavity	1.22E-03	11	12	1	1
**Myelopoiesis of bone marrow**	**3.64E-03**	**2: NPM1, RARA**	**2**	**1**	**0**
**Quantity of trabecular bone**	**4.08E-03**	**2: CREBBP, SMO**	**2**	**1**	**0**
Small indels					
Tissue development	1.19E-03	5	5	1	0
Developmental process of tissue	1.35E-03	5	5	1	0
Development of organ	5.05E-03	4	4	1	0
Organogenesis	5.32E-03	4	4	1	0
Colony formation of tumour cell lines	6.25E-05	3	3	1	0
Colony formation of cells	4.44E-04	3	3	1	0
Colony formation	5.46E-04	3	3	1	0
Developmental process of tumour cells	3.81E-03	3	3	1	0
Colony formation of carcinoma cell lines	5.94E-05	2	2	1	0
Apoptosis of nervous tissue cell lines	2.49E-04	2	2	1	0
**Outgrowth of bone marrow cells**	**7.56E-04**	**1: ALK**	**1**	**1**	**0**
**Inflammatory response of bone marrow-derived macrophages**	**1.26E-03**	**1: RELA**	**1**	**1**	**0**

The sorting is performed by number of genes.

The bold data reflects the processes directly associated to bone.

IVA found 111 genes with 202 germline CD-SNVs associated to cancer (Table 5). Fifteen genes, which had 43 somatic CD-SNVs were associated to “bone marrow cancer and tumours”. In the case of small indel, all six genes, with a finding, are associated to cancer and the found small indels are all somatic. The disease named as “tumourigenesis of bone tumour” was associated to small indel in *ALK* gene and was present only in the tumour tissue.

**Table 5:** **The diseases associated to CD-SNVs and small indels**

**Disease name**	***p*** **value**	**Number of genes associated**	**Number of variances found**	**Tumour tissue**	**Control tissue**
CD-SNVs					
Cancer	7.04E-23	111	202	1	1
Tumourigenesis	8.21E-16	111	202	1	1
Cancers and tumours	3.37E-15	111	202	1	1
Organismal injury and abnormalities	9.45E-17	105	194	1	1
Carcinoma	3.46E-25	99	186	1	1
Solid tumour	2.64E-24	99	186	1	1
Epithelial neoplasia	3.34E-23	99	186	1	1
Epithelioma	3.34E-23	99	186	1	1
Breast or colorectal cancer	5.45E-23	83	164	1	1
Malignant neoplasm of abdomen	6.93E-20	83	169	1	1
**Bone marrow cancer**	**1.69E-03**	**15: CREBBP, EPHA2, FGFR2, KCNJ12, KMT2C, LILRB3, MUC17, MUC4, MYBPC3, NPM1, RARA, SMO, TCF3, TTN, TUBG1**	**43**	**1**	**0**
**Bone marrow cancer and tumours**	**1.69E-03**		**43**	**1**	**0**
Small indels					
Cancer	9.07E-03	6	6	1	1^a^
Hematologic cancer	2.36E-04	4	4	1	1^a^
Hematologic cancer and tumours	2.36E-04	4	4	1	1^a^
Hematological neoplasia	8.01E-04	4	4	1	1^a^
Lymphohematopoietic cancer	9.12E-04	4	4	1	1^a^
Disease of colon	7.88E-03	4	4	1	0
Hematological disease	8.15E-03	4	4	1	1^a^
Immunological disease	1.28E-02	4	4	1	1^a^
Gastrointestinal tract cancer	2.00E-02	4	4	1	0
Gastrointestinal tract cancer and tumours	2.02E-02	4	4	1	0
**Tumourigenesis of bone tumour**	**7.04E-03**	**1: ALK**	**1**	**1**	**0**

^a^Here, only one gene PRR23C has a small indel in heterozygous form, which most likely does not affect the gene function. See Table 2.

The bold data reflects the diseases directly associated to bone.

With the osteosarcoma patient’s tumour and control tissue, WES data IVA found six pathways associated to CD-SNVs and six to cancer driver small indels (Table 6). All the mutations considered here were somatic. In the case of CD-SNVs, the statistically most significant association was between tumour and WNT/β-catenin signalling pathway. In the case of small indels, associations with different cytokine pathways were found. Also, a pathway directly linked to the bone tissue—“RANK signalling in osteoclasts” was brought front.

**Table 6:** **The pathways associated to cancer**

**Pathway name**	***p*** **value**	**Number of genes**	**Genes**	**Number of variants**	**Tumour tissue**	**Control tissue**
CD-SNVs						
Wnt/β-catenin signalling	7.07E-04	6	CREBBP, RARA, SMO, SOX10, SOX7, TCF3	6	1	0
Epithelial adherens junction Ssignalling	1.26E-02	4	IQGAP1, KEAP1, TCF3, TUBG1	4	1	0
Germ cell-sertoli cell junction signalling	2.10E-02	4	GSN, IQGAP1, KEAP1, TUBG1	5	1	0
Mouse embryonic stem cell pluripotency	2.59E-02	3	CREBBP, SMO, TCF3	3	1	0
Regulation of the epithelial-mesenchymal transition pathway	3.40E-02	4	FGFR2, SMO, TCF3, ZEB2	4	1	0
Hereditary breast cancer signalling	4.95E-02	3	CREBBP, NPM1, TUBG1	3	1	0
Small indels						
IL-17A signalling in gastric cells	8.79E-03	1	RELA	1	1	0
Role of JAK1, JAK2 and TYK2 in interferon signalling	9.54E-03	1	RELA	1	1	0
Interferon signalling	9.79E-03	1	RELA	1	1	0
IL-15 production	1.00E-02	1	RELA	1	1	0
TNFR2 signalling	1.05E-02	1	RELA	1	1	0
RANK signalling in osteoclasts	2.86E-02	1	RELA	1	1	0

3) **Results from CEQer software**

**Figure 1:**
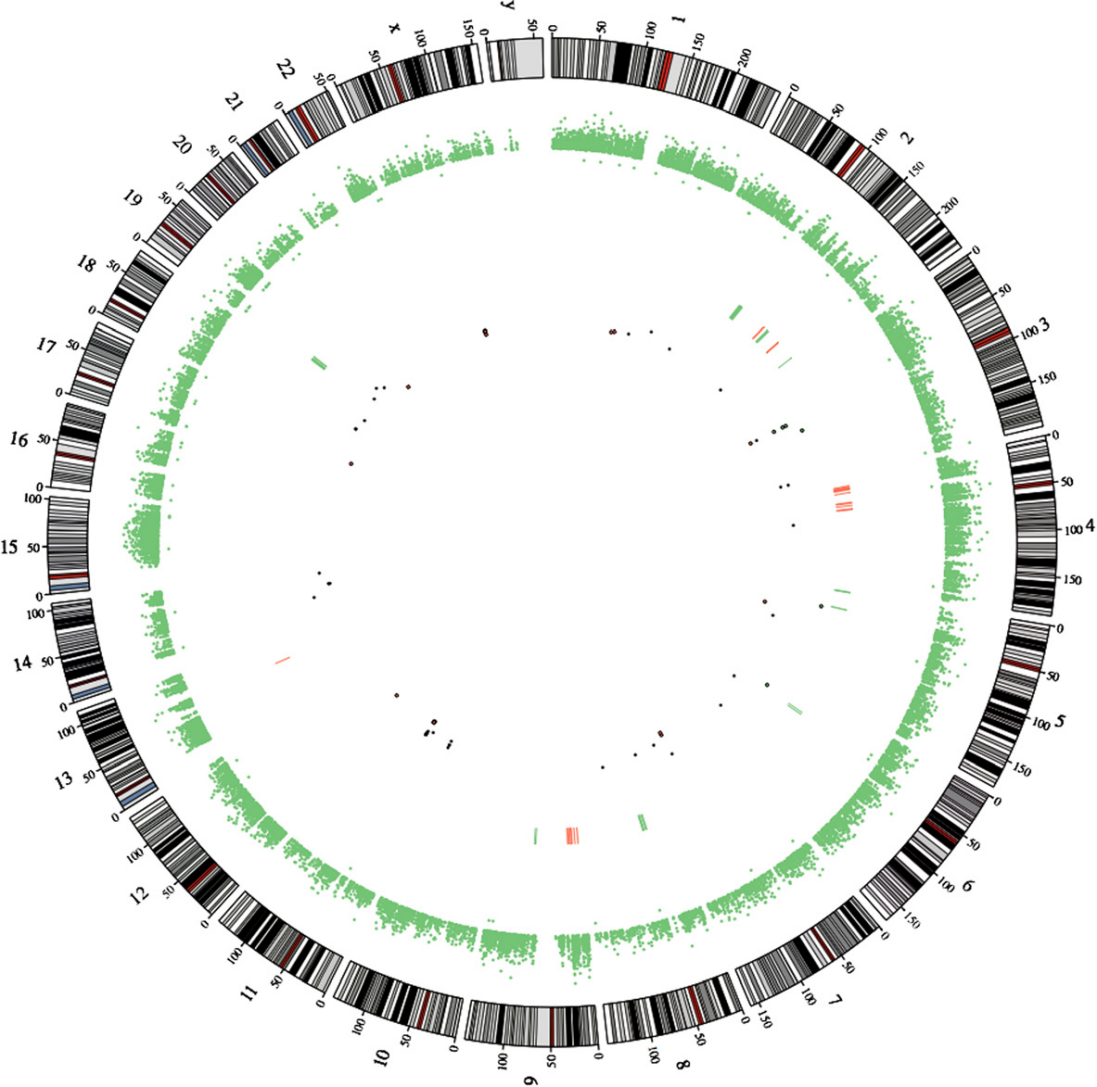
**Circos plot illustrating the CNVs and LOHs in the OS tissue compared to that in the control tissue.** CNVs are marked as lines in the centre: red—gain and green—loss. LOHs are marked as dots in the centre: black—copy neutral, green—copy gain and red—copy loss.

**Figure 2:**
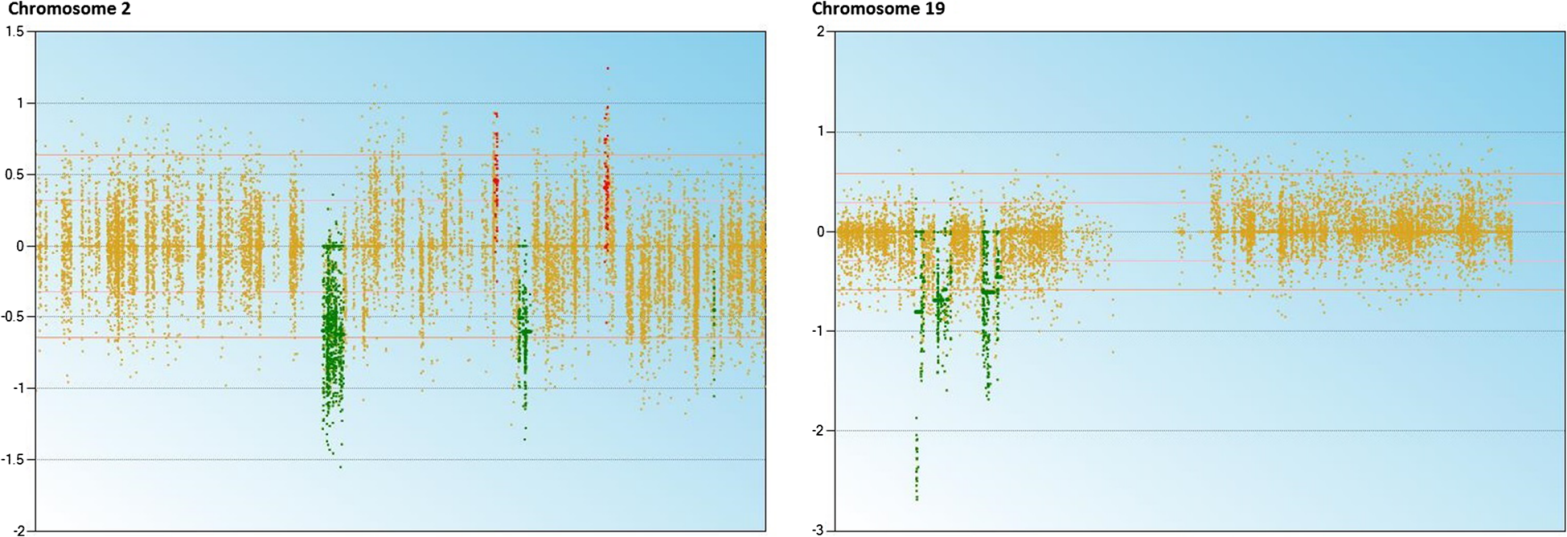
**The CNVs in chromosomes 2 and 19 in the osteosarcoma tissue compared to that in the control tissue.** Data analysis performed with CEQer software.

We applied CS to analyse CNVs in tumour and non-tumour tissue exomes. Compared to the control tissue, in the tumour tissue, the loss of coding sequences was found in 6 chromosomes and 183 genes and gain of coding sequences in 4 chromosomes and 65 genes (Figure 1). The loss or gain of coding sequences was altogether in 8 chromosomes, and the most altered were chromosomes 2 and 19 (193,701 bp and 115,358 bp, respectively; Figure 2). The loss of heterozygosity was detected altogether in 68 regions in 37 genes, located in 15 different chromosomes (Additional file 3).

### Integrative analysis

The integrative analysis narrows down the large list of findings from NGS data. When combining the results from WES data (AS, IVA, CS) and RNA-seq data [10], we found some interesting and rather logical associations, which we would like to emphasize.

### SNVs, small indels and RNA expression

To reduce down the complexity of data we received from AS, we decided to perform as follows. In the case of SNV data, we observed both somatic and germline SNVs, which are homozygous in the tumour tissue and should have an effect on translation (nonsynonymous, stopgain, stoploss findings). Thus, we got 527 homozygous germline SNVs (in 392 genes) and 8 homozygous somatic SNVs (in 7 genes), which are located in genes with altered expression in the tumour tissue compared to that in the control tissue. If also considering the ljb2 database scores, seven homozygous SNVs with high disease-causing probability remained (Table 7).

**Table 7:** **The integrative analysis—genes with altered expression pattern** [10] **and SNVs annotated with ANNOVAR software**

**Gene name**	**Transcript name—exon number: nucleotide change/amino acid change**	**ljb2 score/indel**	**Chr number**	**Start**	**End**	**REF/ALT**	**logFC**	**FDR**
Germline mutations homozygous in tumour tissue								
STEAP4	NM_024636**—**exon2: c.G364A/p.A122T	0.647	Chr7	87913221	87913221	C/T	3.015	1.44E-19
	NM_001205316**—**exon2: c.G364A/p.A122T							
	NM_001205315**—**exon3: c.G364A/p.A122T							
DDX60L	NM_001012967**—**exon18: c.T2491C/p.C831R	0.711	Chr4	169341435	169341435	A/G	2.349	2.67E-14
MT1A	NM_005946**—**exon3: c.A152G/p.K51R	0.785	Chr16	56673828	56673828	A/G	−3.094	0.00795
ACOX1	NM_004035**—**exon7: c.C936G/p.I312M	0.872	Chr17	73949540	73949540	G/C	−0.809	0.01538
	NM_007292**—**exon7: c.C936G/p.I312M							
	NM_001185039**—**exon7: c.C822G/p.I274M							
TMC7	NM_001160364**—**exon6: c.G431A/p.G144E	0.695	Chr16	19041595	19041595	G/A	1.266	0.01726
	NM_024847**—**exon6: c.G761A/p.G254E							
MYO7A	NM_001127179**—**exon27: c.3514_3535del/p.1172_1179del	Frameshift deletion	Chr11	76895771	76895792	GGAGGCGGGGACACCAGGGCCT/-	1.541	0.03810
ATRNL1	NM_001276282**—**exon8: c.1399_1400insTT/p.L467fs	Frameshift insertion	Chr10	116931101	116931101	-/TT	2.321	0.04535
Somatic mutations homozygous in the tumour tissue								
TMEM120B	NM_001080825**—**exon3: c.G274A/p.D92N → X → COSM1599921	0.981	Chr12	122186317	122186317	G/A	−1.548	0.00064
TMEM131	NM_015348**—**exon31: c.C3947T/p.P1316L	0.945	Chr2	98409046	98409046	G/A	−0.799	0.01371
EI24	NM_001007277**—**exon9: c.733dupC/p.R244fs	Frameshift insertion	Chr11	125452300	125452300	-/C	−0.815	0.01569

These germline or somatic SNVs are all nonsynonymous and homozygous in the tumour tissue and according to ljb2 database have a disease-causing effect.

In the case of small indels detected with AS, we observed the somatic and germline indels, which were homozygous in the tumour tissue. There was 52 germline and 26 somatic indels in introns of the genes, which expression pattern has also changed (data not shown). Furthermore, there was five germline and three somatic indels in exons of the genes with altered expression. Thus, we found altogether three frameshift small indels, which possibly have an effect on translation (frameshift insertions and deletion in exons) (Table 7).

In the case of homozygous cancer driver SNVs and small indels found with IVA (Table 2), only four genes have altered expression pattern in the tumour tissue compared to that in the control tissue. The mRNA expression was increased in the case of *PLAT* (log fold change (logFC) = 3.65, false discovery rate (FDR; corrected statistical significance) = 8.27E-27), *AGTPBP1* (logFC = 0.91, FDR = 0.039) and *LRRC37A3* (logFC = 1.14, FDR = 0.0072) and decreased in the case of *SFB1* (logFC = −1.33, FDR = 0.0037).

### CNVs, LOHs and RNA expression

When analysing the CNV results together with RNA expression results, we found that with gained copy numbers, there were altogether 22 genes, with altered expression profile—20 genes with increased and 2 genes with decreased mRNA expression. In the case of loss copy of number, 74 genes’ expression profile had changed—11 genes with increased and 63 genes with decreased mRNA expression. In Table 8, the genes with the lowest FDR values for gene expression results are presented. Here, we would emphasize that the *INSR*, which has copy number loss in area covering 174,552 bp has also a remarkable decrease in mRNA expression (3.36 times; FDS = 9.67E-31). However, there are also several genes with CNVs, which could be associated to cancer.

**Table 8:** **The integrative analysis—CNVs and RNA expression data** [10] **is observed together**

	**CNVs**						**RNA expression**	
**Gene name**	**Chr number**	**Start**	**End**	**Area length**	**CNV** ***p*** **value**	**Copy number fold change**	**logFC**	**FDR**
Loss								
INSR	Chr19	7119459	7294011	174,552	3.18E-11	−6.64	−3.36	9.67E-31
NFIX	Chr19	13106583	13201204	94,621	0	−10.82	−2.45	1.63E-17
FARSA	Chr19	13034964	13044558	9,594	0	−10.82	−2.62	1.96E-16
RAD23A	Chr19	13056627	13063667	7,040	0	−10.82	−2.40	4.46E-16
GINS4	Chr8	41386724	41399418	12,694	8.28E-05	−3.94	−2.79	1.31E-15
GADD45GIP1	Chr19	13064971	13068050	3,079	0	−10.82	−2.82	3.67E-15
IFIH1	Chr2	163123588	163175218	51,630	0	−10.11	2.20	3.69E-14
RPL31	Chr2	101618690	101622885	4,195	0	−9.39	−2.05	5.08E-13
PLEKHG4B	Chr5	156185	181790	25,605	1.08E-05	−4.40	−2.17	1.58E-12
ZNF358	Chr19	7581003	7581135	132	3.18E-11	−6.64	−2.59	2.56E-11
ARHGEF18	Chr19	7459998	7532004	72,006	3.18E-11	−6.64	−1.96	1.44E-10
STX10	Chr19	13255223	13260987	57,64	0	−10.82	−2.55	1.96E-10
COL5A3	Chr19	10102679	10121147	18,468	4.14E-04	−3.53	−1.92	5.45E-10
MGAT4A	Chr2	99242185	99347589	105,404	0	−10.22	1.87	9.95E-10
Gain								
SLC40A1	Chr2	190428309	190428951	642	1.83E-05	4.28	2.22	1.05E-14
KIT	Chr4	55524094	55603446	79,352	1.69E-06	4.79	2.54	1.17E-13
PTPLAD2	Chr9	21008019	21031635	23,616	7.73E-14	7.48	3.02	4.49E-13
ATP8A1	Chr4	42571177	42629126	57,949	1.83E-07	5.22	2.65	4.54E-10
FOCAD	Chr9	20658308	20993327	335,019	7.73E-14	7.48	1.94	9.12E-08
FAM200B	Chr4	15683351	15692070	8,719	3.54E-05	4.14	1.83	8.59E-07
SLIT2	Chr4	20255234	20512189	256,955	4.16E-05	4.10	1.35	1.73E-05
MLLT3	Chr9	20353522	20622514	268,992	7.73E-14	7.48	1.94	2.19E-05
LCORL	Chr4	17887690	18023483	135,793	4.16E-05	4.10	1.40	5.04E-05

Only the genes with lowest FDR value are presented.

Combining the LOH and mRNA expression data, we found that in the tumour tissue, the expression of four genes with LOH has increased significantly and expression of five genes with LOH has decreased significantly (Table 9). The rest of the genes with LOHs had no significant changes in mRNA expression level, and two genes were not detected with RNA-seq (*FLJ20518*, *MANSC4*) [10].

**Table 9:** **The integrative analysis - loss of heterozygosity and RNA expression data observed together**

**Gene name**	**Chr number**	**LOHs**				**RNA expression**	
		**LOH position**	**Alleles**	**LOH**	**LOH** ***p*** **value**	**logFC**	**FDR**
MS4A14	Chr11	60165358–60165379	G/C	CopyNeutralLOH	0.025	2.46	3.20E-08
DSC2	Chr18	28666554–28666556	A/C	CopyNeutralLOH	0.025	1.87	3.82E-07
RPS4X	ChrX	71495409–71495414	G/C	CopyNeutralLOH	0.01	−1.44	7.25E-07
RPS23	Chr5	81571874	A/C	CopyNeutralLOH	0.005	−1.43	1.04E06
IL7R	Chr5	35874575	C/T	1AlleleGain	0.025	1.59	6.69E-06
PCNXL2	Chr1	233398713	C/T	CopyNeutralLOH	0.01	1.20	0.00027
HILPDA (C7orf68)	Chr7	128098270	T/G	CopyNeutralLOH	0.0001	−1.12	0.00094
HRNR	Chr1	152188041	C/T	Allele(s)Loss	0.025	−3.09	0.00796
MUC4	Chr3	195515594, 195516630	C/G	CopyNeutralLOH	0.025	−2.22	0.01230

Only the genes with significant mRNA expression changes in the tumour tissue compared to that in the control tissue are presented.

For additional information, please see the supplementary material as separate files for AS, IVA and CS combined with RNA-seq data.

## Discussion

In this study, the exome profiles of the osteosarcoma patient’s tumour and normal bone tissue were compared. Additionally, the RNA-seq data from our previous work was used [10]. For WES data analysis, several softwares were applied and possibly some of them are better in detecting some mutations and not so effective in detecting others. Still, we think it is more beneficial to use different approaches and we believe it is easier to follow, if we discuss separately the results gained from each software.

The ANNOVAR software annotated a large amount of genes with SNVs and small indels, applying refGene hg19 database. Over 2,700 somatic SNVs and small indels were detected specifically in the tumour tissue, from which almost 300 are homozygous. These findings are located all over the exome. This demonstrates that the changes in OS genome are not concentrated into a single or few areas but are rather distributed.

When using ljb2 database, AS detected four homozygous somatic mutations in the tumour tissue, which could potentially cause a disease. These nonsynonymous mutations were located in *ESX1*, *CDC27*, *TMEM120B* and *TMEM131*. Additionally, in the case of *TMEM120B* and *TMEM131*, the mRNA expression has decreased substantially in the tumour tissue compared to that in the control tissue [10]; however, further studies are needed to confirm the possible associations between found mutations and gene expression level. Available data about the possible associations between OS and these genes is very limited. In *TMEM120B*, a gene with an unclear function, the mutation COSM1599921 has been previously detected in glioma [33]. The *CDC27* is a gene possibly controlling the timing of mitosis and may have an important role in tumour cell division [34]. In addition to the somatic mutation, the *CDC27* had 33 heterozygous germline disease-causing mutations (nonsynonymous) (data not shown). In the case of breast cancer, the *CDC27* has been demonstrated to be a promising biomarker in predicting the disease progression and prognostication [35]. Thus, these somatic mutations may have some effect on OS pathogenesis. Especially the abundant changes in *CDC27* may be important in terms of regulating OS tumour cell division.

In the tumour tissue, we detected homozygous somatic small indels causing the frameshift in five genes—*EI24*, *ALG1L2*, *TIGD6*, *GPATCH4* and *SSPO*. None of these genes have previously been associated to OS, and according to our RNA-seq data, only *EI24* of these five genes has altered mRNA expression—it has decreased in the tumour tissue [10], which could be due to the insertion in exon 9. The *EI24* encodes a tumour suppressor and is an immediate-early induction target of TP53-mediated apoptosis—it binds to antiapoptotic BLC2. Furthermore, the *EI24* has found to be highly mutated in the case of aggressive breast cancer and is rather associated to tumour invasiveness than development of the primary tumour [36-38]. In the present case, we found no mutations in *TP53* nor was the expression altered [10]; thus, according to this data, we may suggest that the TP53 is functional in the tumour tissue. However, the TP53 pathway may still be suppressed due to mutated and downregulated *EI24*. Moreover, the aggressive nature of OS is correlated to this finding.

Appling Ingenuity Variant Analysis software, we found over 200 cancer driver variants and 93% of these possibly cause the loss of gene function. Thirteen homozygous somatic CD-SNVs were detected in different genes—*RGPD3*, *PRDM9*, *FOXK1*, *CCZ1*, *PLAT*, *AGTPBP1*, *SARDH*, *FAH*, *CDC27*, *SBF1*, *LRRC37A3*, *ARL17A* and *LILRB3*. The mRNA expression of *PLAT*, *AGTPBP1* and *LRRC37A3* has increased and of *SFB1* has decreased significantly [10]. We found no previous data about the associations between OS and these genes, except *SBF1*. With previous OS studies, another missense mutation (p.E1539K) has detected in *SBF1* [39]. SBF1 is a SET (a nuclear oncogene) binding factor 1 and may inhibit the cell division [40]. The decreased expression in the tumour tissue may be responsible for the increased cell proliferation. Some other associations, which might be interesting—*PLAT* gene is important for cell migration and tissue remodelling and the overexpression might cause hyperfibrinolysis [41], which has not previously described in the case of OS. Two mutations in *ARL17A* have detected in chondrosarcoma cells [42]. In the case of *CDC27*, the same mutation (p.E6G) was also brought front by AS as potentially disease causing, which is discussed above. Thus, it is highly likely that at least some of these genes participate in some level of OS pathogenesis.

Additionally, with IVA four homozygous somatic small indels were detected in the tumour tissue. These were in noncoding regions of genes *CTCFL*, *PRR23C*, *CDCA7L* and *ALK*; thus, the effect might be post-transcriptional. *CTCFL* is a genetic paralog of *CTCF*; latter is an important methylation pattern regulator. In the case of *CTCF*, it has previously demonstrated that in the OS tissue, the changes in its methylation pattern may also cause loss of imprinting of *IGF2* and *H19* genes, which further alters their expression pattern [43]. In our OS patient’s tumour tissue, the mRNA expression of both *IGF2* and *H19* has increased significantly (FDR = 3.46E-15 and FDR = 0.0015, respectively) [10]. Thus, the association may be valid here also. In *PRR23C*, one missense mutation (p.R190W) has detected previously in the OS tissue [42]. *ALK* encodes a receptor tyrosine kinase and is rearranged, mutated or amplified in several tumours. However, in the case of OS, there are only few reports about ALK [44,45]. In addition, two heterozygous somatic small indels were detected in *DSPP* and *RELA* exons; however, we found no previous data about these findings and associations to OS. The small indels might have an effect on the expression of these genes both pre- and post-transcriptional level; however, these suggestions need to be further studied.

According to IVA, there were several genes with more than one mutation—in *MUC4*, there were even 22 somatic mutations in exons and 44 in introns, although they all were heterozygous. Thus, we found *MUC4* locus to be the most altered in the tumour tissue compared to that in the control tissue. This might explain why its mRNA expression in the tumour tissue has decreased (FDR = 0.012) [10]. Mucin 4 is among major constituents of mucus, and it has demonstrated that primary bone tumours rarely express MUC4 protein [46], which correlates to our finding. Furthermore, with IVA, we found mucin complex (*MUC2*, *MUC4*, *MUC6*) to have a highest significance in OS among others. However, there are also other mucin genes (MUC16, MUC17, MUC20) with somatic heterozygous CD-SNVs. The expression pattern of all other detected mucins has not changed significantly. Thus, mucins may have a role in OS pathogenesis, but we dear not to make any further conclusions.

With IVA, there was four bone-related processes brought front only in the case of the tumour tissue—“myelopoiesis of bone marrow” (*NPM1*, *RARA*), “quantity of trabecular bone” (*CREBBP*, *SMO*), “outgrowth of bone marrow cells” (*ALK*) and “inflammatory response of bone marrow-derived macrophages” (*RELA*). Furthermore, in disease list, 16 genes with over 40 somatic variations were associated to “bone marrow cancer” and “bone tumour”; however, there were also over 200 germline CD-SNVs associated to cancer. Thus, here, we would like to emphasize that in the case of both cancer-associated processes and diseases, the ones associated with bone are somatic mutations; however, the findings possibly promoting cancer are germline mutations. This is one of the phenomena, which we would like to observe in our future studies.

The most significant pathway found with IVA was “WNT/β-catenin signalling pathway” (altered genes: *CREBBP*, *RARA*, *SMO*, *SOX10*, *SOX7*, *TCF3*). Reviewed in [15], the pathway is required for bone development and has demonstrated to be altered in pathogenesis of OS—overexpression of numerous WNT pathway components including WNT ligands, FZDs and LRP receptors and epigenetic silencing of the pathway inhibiting genes, i.e. *WIF1*. However, in our previous study, we found *WNT7B* and *WNT11* to be downregulated and *WNT2B* and *WNT5B* upregulated; *FZD4* and *FZD8* upregulated and *LRP8* and *LRP12* downregulated and *DVL3* downregulated and *WIF1* and *SOST* upregulated. Additionally, genes with CD-SNVs—*RARA*, *SMO* and *SOX7* were upregulated [10]. Thus, our results are rather controversial to several previous studies demonstrating the WNT/β-catenin pathway to be upregulated [47-49]. However, there are also studies correlating to our findings [50,51]. As our study is based on a single case, we dear not to conclude, why the WNT/β-catenin pathway is rather downregulated here, but we suggest the controversial results may occur due to major heterogeneity of OS. Nevertheless, the present study demonstrates that in addition to altered expression patter, the genes involved in WNT/β-catenin signalling pathway carry the CD-SNVs.

In the case of small indels, the IVA brought front the pathways associated to RELA and these are mostly cytokine signalling pathways (Table 6). Previously, it has demonstrated that interaction of IL17A and IL17AR promotes metastasis in OS cells. Furthermore, IL17 stimulates osteoclast resorption [52]. In our previous study, we found IL17AR to be significantly upregulated [10]. Osteoclasts are important in pathogenesis of OS—the more active they are, the more aggressive the tumour is [53]. RELA is demonstrated to enhance the osteoclast differentiation [54]. As IVA predicts the loss of RELA functionality (at least partially, as the small indel is heterozygous), in the present case, the OS might not have been as aggressive as it usually would.

Previously, it has demonstrated that chromothripsis event is common to early stage of OS—hundreds of genomic rearrangements will appear in a single instability event [26]. In the present case, the CEQer software detected nearly 2,400 gain and loss events in 8 chromosomes involved, which should qualify as the chromothripsis. However, the initiating cause of this massive rearrangement is unknown, as there were no traumas or other environmental causes we are aware of.

In general, the gain of copy number should increase the mRNA expression and loss of copy number should decrease the expression [6]. In present work, this pattern was valid in the case of 86.5% of the genes with CNVs and altered expression. One of the strongest findings here was the amount of CNVs in *INSR*, which expression has decreased remarkably (Table 8). The main physiological role of the insulin receptor appears to be metabolic regulation [55]. However, together with IGF1R it forms a hybrid receptor for IGF1, latter together with IGF2 is thought to have a key role in driving the proliferation and survival of sarcoma cells [56]. Furthermore, the growth hormone and IGF1 axis controls the growth and bone modelling/remodelling [57]. Additionally, the IRS1, which is phosphorylated by the INSR, is important for both metabolic and mitogenic pathways [58]. In the present case, the mRNA expression of both *IGF1* and *IGF2* has increased (FDR = 4.65E-35 and FDR = 3.46E-15, respectively); however, the expression of *IGF1R* remained the same in the tumour tissue compared to that in the control tissue [10]. Furthermore, in *IGF1R* we found a heterozygous germline nonsynonymous mutation (p.G1117R) with AS, which according to ljb2 database is a disease causing (data not shown). Similarly to *INSR*, the mRNA expression of *IRS1* is decreased in the tumour tissue compared to that in the control tissue (FDR = 2.62E-10) [10]. Thus, in the present case it seems, the proliferation of tumour cells might be rather supported by increased effect of IGF1, IGF2 and IGF1R homodimer associations, than IGF1, IGF2 and INSR-IGF1R heterodimer associations or INSR effects on IRS1.

The loss of heterozygosity has been reported to be extensive in OS exomes [39]. In the present case, we did not detect whole chromosome or gene region loss; however, we did detect the loss of heterozygosity in smaller regions. The genes with LOH findings and increased mRNA expression—*MS4A14*, *DSC2*, *IL7R* and *PCNXL2* have not associated to OS previously. However, in the case of *DSC2*, the overexpression has demonstrated to be inversely correlated to bone metastasis-free survival [59]. The mutations in *IL7R* exon 6 have been demonstrated to be present in leukaemia patients’ bone marrow samples but not associated to other solid tumours [60]. The five genes with LOHs and decreased mRNA expression—*RPS4X*, *RPS23*, *HILPDA* (*C7orf68*), *HRNR* and *MUC4* also do not have previous information associated to OS. Nonetheless, also the LOH analysis brought forward different genes in mucin family. In addition to *MUC4*, there were also other genes with LOHs but with insignificant mRNA expression changes in the tumour tissues—*MUC2*, *MUC6* and *MUC17*. Thus, these results also support the idea that mucins might have a role in pathogenesis of osteosarcoma.

In summary, the present case has several characteristics previously demonstrated in OS. The wide genomic arrangements have appeared—SNVs and small indels all over the genome and CNVs in some chromosomes; and in several cases, these rearrangements may have an effect on gene expression. Furthermore, the germline mutations seem to be associated to cancer in general and somatic mutations to bone tumours. The most significant pathway was the one probably most thoroughly studied in the case of OS—the WNT/β-catenin signalling pathway. We found several genes in this pathway carrying the cancer driver variances. Additionally, the IGF1/IGF2 and IGF1R homodimer signalling might have an essential effect on OS pathogenesis. Which also needs to be emphasized is that according to our data (based on DNA and RNA studies), there is no evidence of a nonfunctional *TP53*; however, the TP53 pathway might be suppressed in further levels—the downregulation of *EI24*. In addition, with this study, we found associations between different genes and OS pathogenesis, which have not demonstrated before in earlier studies. We found the *MUC4* locus to be the most altered in the tumour tissue compared to that in the control tissue; furthermore, several other mucin genes are also possibly associated to OS. The somatic mutation in *CDC27* was brought front by two different data analysis softwares and might have a role in OS pathogenesis.

## Conclusions

All genes, in which the mutations were detected, may be considered as potential targets for additional studies (i.e. functional, histopathological, clinical studies) for finding OS biomarkers. The present study brought front the WNT pathway genes, IGF1/IGF2 and IGF1R homodimer signalling pathway genes, *TP53* together with *EI24*, *MUC4* together with other mucin genes and *CDC27* as potential biomarkers for OS. Finally, as this study is based on a single case and only DNA and RNA analysis, these data may not be taken as conclusive evidence and further studies are needed to confirm the present findings.

## Additional files

Additional file 1:
**ANNOVAR software.** The file contains the list of SNVs (coverage at least 20 times) and small indels detected from WES study. Additionally, the dbSNP135, dbCOSMIC67, ljb2 scores and RNA-seq information is added if available. Additional file 2:
**Ingenuity Variant Analysis software.** The file contains the list of SNVs, which according to IVA are associated to cancer. Additionally, the dbSNP135, SIFT and POLYPHEN functions and RNA-seq information is added if available. Additional file 3:
**CEQer software.** The file contains the list of genes where CNVs and LOHs were detected. Additionally, the RNA-seq information is added if available.
